# Raising Awareness about Sex Trafficking among School Personnel

**DOI:** 10.3390/ijerph21080978

**Published:** 2024-07-26

**Authors:** Elena Savoia, Amy Liu, Amy Leffler, Léa Kay Nadril Churchill, Maxwell Su

**Affiliations:** 1 Department of Biostatistics, Harvard T.H. Chan School of Public Health, 677 Huntington Avenue, Boston, MA 02115, USA; amyliu@hsph.harvard.edu (A.L.); masu@hsph.harvard.edu (M.S.); 2 Community Safety Evaluation Lab, Emergency Preparedness Research Evaluation & Practice (EPREP) Program, Division of Policy Translation & Leadership Development, Harvard T.H. Chan School of Public Health, 677 Huntington Avenue, Boston, MA 02115, USA; 3 U.S. Department of Homeland Security, Washington, DC 20394, USA

**Keywords:** human trafficking, teachers, school counselors, training, videos, awareness

## Abstract

Background: We aimed to (1) understand the level of knowledge about sex trafficking of minors among school personnel and the determinants of such knowledge and (2) test the efficacy of short educational videos in increasing knowledge (awareness level) about sex trafficking of minors among school personnel. Methods: We employed an online survey to gather responses from 741 school personnel living in the US. The McNemar test was used to test for differences in knowledge before and after exposure to the videos. Logistic regression was used to identify predictors of knowledge based on the respondents’ characteristics. Results: Predictors of knowledge about sex trafficking were years of experience in working with youth, level of education, and being a female. Exposure to the educational videos improved school personnel’s basic knowledge about this crime and interest in seeking additional educational material. Conclusion: School personnel have a high level of awareness of risk factors for sex trafficking but less awareness of the definition of sex trafficking in children. Exposure to short educational videos can increase awareness in the short term. There is a need to develop more comprehensive training initiatives for school personnel on sex trafficking. However, training alone is not sufficient, and there is also a need for developing school protocols and programs to provide adequate support to victims of this crime.

## 1. Background

Human trafficking remains a pervasive crime both within the United States and globally. Global prevalence estimates suggest that rates of human trafficking are increasing [1]. In the United States, it is estimated that 80% of those in the commercial sex industry became involved before the age of 18, with the average age for girls of 13 and boys of 12 years old [2]. To describe the devastating impact on society of this phenomenon, Perk et al. deemed child trafficking as a “pandemic of human rights violations” [3].

Under federal criminal law (18 U.S. Code 1591), child sex trafficking includes a variety of acts such as recruiting, transporting, and advertising that lead a person under the age of 18 to engage in a commercial sex act. In the United States, by law, a minor under 18 cannot legally consent to a commercial sex act, and any instance of commercial sex with a minor is considered child sex trafficking. A commercial sex act is defined as any sex act performed in exchange for anything of value [4].

Combatting such a nuanced crime is inherently complex as the crime feeds on an individual’s vulnerabilities and social power imbalances. A recently published literature review identifies relevant risk factors and vulnerabilities for children’s victimization into sex trafficking including child abuse and neglect, caregiver stress, running away, or being abandoned, among others [5].

The U.S. Department of State calls for increased awareness of sex trafficking, stating that with an increased understanding of this crime, society will be able to better recognize victims and support survivors [6]. Building awareness across avenues and professional roles is a crucial step in adopting a prevention model to combat this crime across geographical boundaries [6].

One pivotal avenue to increase awareness is within the school system. Research on survivors of human trafficking indicates that they did not feel “seen” while in school and that their behaviors were overlooked or discounted [2]. Such behaviors included frequent visits to the school nurse’s office for pains that were discarded or incorrectly labeled as menstrual pains, school absences, poor grades, or being in possession of hotel pens or keys. Schools are a prime location to train and educate personnel in recognizing potential victims of human trafficking because children spend most of their day at school and have many interactions with teachers and school personnel in general. School counselors, due to their training and daily interaction with youth, are in a unique position to recognize situations of abuse, potential indicators of human trafficking, and the related needs of the victims [7]. Recent research emphasizes the role of training in increasing awareness of sex trafficking and improving self-efficacy among children for combatting this crime [8]. However, as described by Rizo et al. [9], who surveyed 76 principals in North Carolina, there are limited training opportunities for school personnel to learn about sex trafficking and mechanisms to respond to potential cases. Human trafficking awareness training tailored to professionals who regularly interact with children needs to enable them to recognize the nuance and range of behaviors associated with the victimization process. Such training should include information on how to recognize risk factors that make children more vulnerable to falling into situations of exploitation (e.g., lack of self-esteem) as well as physical and mental health signs of abuse (e.g., bruises, tattoos, anxiety) [10,11]. As noted by Gerassi et al. [12], it is important that training materials include a diversity of case examples based on specific contexts and that the person interacting with the victim is supporting and advocating on their behalf. Yet, there remains a lack of evidence on the impact of such programs, not only on lasting knowledge gained by the individual but also on the ability to translate it into actions that support the victim and avoid bias based on race, socio-economic level, and other individual’s characteristics [6]. To help fill this gap, we aimed to understand the level of knowledge of school personnel about this crime and the efficacy of specific short training interventions. More specifically, we aimed to (1) understand the level of knowledge about the sex trafficking of minors among school personnel and the determinants of such knowledge and (2) test the efficacy of short educational videos in increasing awareness about the sex trafficking of minors among school personnel.

## 2. Methods

For aim 1, we utilized a cross-sectional study design and incorporated both closed and open-ended questions in an online survey using SurveyMonkey Inc. For aim 2, we utilized a pre-post survey design where participants were asked questions before and immediately after watching two educational videos embedded in the survey instrument as well as at a 6-month follow-up. The study protocol and survey questions were approved by the Harvard T.H. Chan School of Public Health Institutional Review Board. Participants consented to participate in the survey prior to responding to the questions. The survey questions were adapted from a previous survey implemented by the research team [13]. For questions related to risk factors and signs associated with human trafficking, we referred to the Blue Campaign resource guide for school officers [14]. For this study, the questions underwent additional cognitive testing on nine individuals (students and university staff not involved in the study) before implementation, and revisions were made accordingly.

### 2.1. Participants

We recruited school personnel and used two mechanisms to identify the respondents. First, we purchased two panels of survey respondents, one from Pollfish (*n* = 550) and another one from Prolific (*n* = 100) and used a screening question with job categories to select people working as school personnel. Then we purchased a dataset with the names of public high schools in the US from the National Center for Education Statistics (based on 2020–2021 data) and extracted a random sample of 1500 schools. For each school, we manually searched for the school counselor’s name and email on the school website. We invited each counselor to participate in the survey via email, to which 91 people responded. If contact information was not available, no further attempt was made to contact the school to obtain the counselor’s e-mail. This method was used to increase the number of school counselors because this professional role was underrepresented in both the Pollfish and Prolific panels.

Both Pollfish and Prolific use crowdsourcing technology [15,16]. However, Prolific assigns an ID to each respondent making it possible to survey the same individual over time, a feature not available through Pollfish at the time of our study. As such, the Prolific panel and the school counselors’ emails, manually identified by our team, were used to create a sub-cohort of teachers and school counselors for the 6-month follow-up. The surveys were implemented between November 2022 and January 2023.

### 2.2. Video Intervention

The videos tested as part of this study included two Blue Campaign videos focused on human trafficking regarding minors, each approximately two minutes long, entitled (1) Mia’s story—an animated story of a girl victim of human trafficking who was groomed online and coerced into sex trafficking and (2) The teacher—an animated story describing a teacher who noticed a change in Mia’s school performance and suggested that the girl talk to the school counselor. The two videos are part of the Blue Campaign, a campaign developed by the U.S. Department of Homeland Security (DHS) to raise awareness about human trafficking, and are available to the public from the DHS media library [17].

### 2.3. Dependent Variables

Table 1 depicts the survey questions and coded answer options used to define five dependent variables to describe different types of awareness.

### 2.4. Independent Variables

School personnel who participated in this survey were working in high schools. Their socio-demographic variables are described in Table 2. We used the zip code information, reported by each respondent, to describe the characteristics of the geographic area where the respondent lives. Data from two different sources were used to obtain characteristics of the population within the participants’ geographic area. Census region (Midwest, Northeast, South, and West), median household income, percentage of the population reporting as of white race, and percentage of the population under the 1.37 of the poverty threshold were derived from the Agency for Healthcare Research and Quality’s (AHRQ) database on Social Determinants of Health (SDOH) 2020 zip code data [18]. The density of the population was derived from the data resource hub Standard Co., Atlanta, GA, USA [19].

In addition, we asked respondents about their knowledge of their school protocol (if any) to report potential cases of sex trafficking, if they ever reported a case, and if they ever received training on this topic.

### 2.5. Data Analysis

#### 2.5.1. Descriptive Statistics

Descriptive statistics were computed to describe the respondents’ socio-demographic characteristics. The sample was also described in terms of perceived knowledge, past training experience, awareness of potential risk factors and signs of human trafficking, and reporting mechanisms.

#### 2.5.2. Multiple Factor Analysis (MFA)—Extension of Principal Components Analysis (PCA)

MFA [20,21] was used to test the dimensionality of multi-item scales for the questions describing awareness of situations experienced by youth indicative of potential risk factors for sex trafficking (outcome variable #2 in Table 1) and signs of sex trafficking (outcome variable #3 in Table 1).

MFA extends the principles of PCA to accommodate multiple datasets that capture when several sets of variables have been measured on the same set of observations. In this case, the questions describing awareness of situations experienced by outcome variable #2 and outcome variable #3 in Table 1 were measured pre- and post-video on the same participants. For each scale, we retained principal components in the MFA that corresponded to eigenvalues greater than 1 (Kaiser criterion) [22] and dropped any items with factor loadings less than 0.4, which are commonly used cutoffs in factor analysis. In addition, to test the internal consistency of each multi-item scale, Cronbach’s alpha [23] was calculated for each scale pre- and post-intervention. For the retained components, scores were created as the matrix product of the survey responses and the factor loadings from the aggregated dataset.

#### 2.5.3. Pre–Post-Exposure Assessment

To assess the impact of the exposure to the videos on the pre-identified outcome variables, we compared participants’ responses before watching the videos (baseline survey data) with (1) immediately after watching the videos and (2) at the 6-month follow-up using McNemar’s χ^2^ Test and the Wilcoxon signed-rank test. All statistical tests were performed at the 5% alpha level using the software RStudio Version 2023.03.0+386.

#### 2.5.4. Multivariable Models

Using the survey data gathered at baseline, we conducted logistic regression models using the outcome variables reported in Table 1 to identify determinants of knowledge about human trafficking including the following independent variables: age, sex, race, experience, level of education, job type, median household income (area level), percentage of the population reporting as of white race (area level), percentage of the population under the 1.37 of the poverty threshold (area level), the density of the population (area level), and prior attendance to training on sex trafficking. To determine the most appropriate representation for predictors, age, experience in working with youth, and level of education, likelihood ratio tests (LRTs) were used to evaluate whether they exhibited a better fit as ordinal variables or as categorical variables. The goodness-of-fit of the final models was tested using the Hosmer–Lemeshow test [24].

#### 2.5.5. Coding of Open-Ended Questions

Respondents were asked two open-ended questions to gather (1) their input on what situations could put youth at risk of sex trafficking and (2) in the event the respondent had reported a potential case of sex trafficking in the past, what prompted them to report it. The two questions were analyzed by manually coding and grouping the answers into themes using a deductive approach. Using such approaches, both researchers first met to define the codes; subsequently, one researcher performed the coding first and a second researcher reviewed the coding to confirm the fit of the excerpts with the pre-determined codes, and any discrepancy between the two researchers was discussed and solved to achieve consensus on the main themes derived from the analysis and their interpretation.

## 3. Results

### 3.1. Sample Characteristics

Table 2 describes the sample characteristics. Survey data include 741 respondents: Public school sample (*n* = 91), Pollfish (*n* = 550), and Prolific (*n* = 100). All 741 respondents answered questions before watching the videos and right after. Out of these 741 respondents, for 191 participants, it was possible to administer a follow-up survey at 6 months. This sample corresponds to the Prolific and school sample datasets combined. As explained in the methods, the Pollfish sample was excluded from the 6-month analysis because it does not allow for follow-up surveys (anonymous answers). Out of these 191 individuals, 120 (62.8%) responded to the 6-month follow-up survey. The sample was predominantly white (77%), female (62%), and under the age of 44 years (63.8%). Educators/teachers made up 61% of the respondents followed by counselors (16%), other school staff (11%), administrators (9%), and mental health professionals (3%).

### 3.2. Multiple Factor Analysis

The multiple-factor analysis for the dependent variables named “Awareness of situations that could potentially put students at risk of becoming a victim of sex trafficking” (variable labeled: risk factors) and “Awareness of situations experienced by students that could potentially be signs of sex trafficking” (variable labeled: signs) (Table 1) led to one component associated with an eigenvalue greater than 1 for each scale, which accounted for 53% and 59% of the variability in the items of the risk factor and signs scales, respectively. For the risk factor scale, factor loadings were between 0.59 and 0.80 with Cronbach’s alpha being 0.80 and 0.90 for pre- and post-intervention responses, respectively. The signs scale had factor loadings between 0.65 and 0.73 with a Cronbach’s alpha of 0.81 and 0.87 for pre- and post-intervention data, respectively. The high factor loadings and internal consistency as measured by Cronbach’s alpha meant it was reasonable to use the first component scores as a measure of the risk factor and signs scales. Final scale scores were calculated using matrix multiplication of the survey results and the factor loading.

### 3.3. Experience in Reporting Potential Cases and Past Training

At baseline, more than half of respondents (56.5%) reported that they had never received training on sex trafficking. Most respondents (91.8%) said they had never reported a potential case of sex trafficking during their lifetime, while 8.2% said they did. Six months after the exposure to the videos, 4.2% said they encountered potential instances of sex trafficking involving students since they watched the videos (6-month timeframe).

At baseline, regarding the existence and use of school protocols to report potential cases of sex trafficking, 48.3% said their school has a protocol and that they know how to implement it, 30.1% said that either they never read the protocol or they have read it but would not know how to implement it, 21.3% indicated that their school does not have a protocol, and the remaining 0.3% said the question was not applicable to their school/work situation. After recoding the variable to represent a binary response indicating whether participants read the protocol and understood how to implement it, we found a 10% increase from the baseline survey to the 6-month follow-up for participants who responded, “I read the protocol, and I know how to implement” (χ^2^ = 4.03, df = 1, *p* = 0.045).

Additionally, at the 6-month follow-up, we asked respondents whether, since watching the videos, they took steps to educate themselves about trafficking. The majority (61.7%) reported taking the following steps: 25.8% (*n* = 31) talked with colleagues about trafficking, 23.3% (*n* = 28) completed some training, 21.7% (*n* = 26) searched for information about human trafficking online, 16.7% (*n* = 20) watched additional videos, and 16.7% (*n* = 20) did some readings about the issue. In contrast, 38.3% (*n* = 46) of participants responded that they did not take any action to educate themselves.

### 3.4. Impact of the Exposure to the Videos on Awareness about Human Trafficking

#### 3.4.1. Face Validity of the Videos

In direct response to both videos, participants were asked (1) whether they think the video (Mia’s story) describes a realistic scenario that one or more of their students could experience and (2) whether they believe the video (The Teacher) describes a situation they could experience as school personnel. In response to the first video, 85.8% said the scenario was realistic, and 87.3% said the second video was realistic as well.

#### 3.4.2. Awareness That Commercial Sex Involving Minors Is a Form of Sex Trafficking

Respondents were asked, both before and after watching the videos, whether they thought that a person under the age of 18 engaging in sexual activity in exchange for money or goods is an instance of human trafficking.

Immediately after watching the videos, from the total of 741 respondents, we observed a significant increase from 57.4% to 80.3% in the percentage of respondents giving a correct answer (χ^2^ = 127.5, df = 1, *p* < 0.001).

Of the 120 participants who completed pre- and post-video surveys and the 6-month follow-up survey, 59.2% of respondents gave a correct response pre-video, 92.5% gave a correct response post-video, and 78.3% gave a correct response at the 6-month follow-up. So, from post-video to the 6-month follow-up, the percentage of respondents giving a correct answer decreased from 92.5% to 78.3% (χ^2^ = 12.19, df = 1, *p* < 0.001), but this was still 20.9% greater compared to the baseline of 59.2% (χ^2^ = 10.30, df = 1, *p* = 0.001).

#### 3.4.3. Awareness of Being in a Position at Work to Interact with Students Who May Be Victims of Sex Trafficking

Respondents were asked, before and after watching the videos, if they felt they could find themselves in a position to encounter students who could be victims of sex trafficking. Out of the 741 total respondents, the percentage of respondents perceiving they could find themselves in that position increased from 67.7% to 81% after watching the videos (χ^2^ = 67.21, df = 1, *p* < 0.001).

For the 120 participants who completed pre- and post-video surveys and the 6-month follow-up survey, 74.2% of the 120 respondents gave a correct response pre-video, 85.8% gave a correct response post-video, and 74.2% gave a correct response at the 6-month follow-up. At the 6-month follow-up, compared to post-video, there was a decrease in correct responses from 85.8% to 74.2% (χ^2^ = 5.63, df = 1, *p* = 0.018), essentially nullifying the effect of the exposure to the videos.

#### 3.4.4. Awareness of Situations That Could Potentially Put Students at Risk of Becoming a Victim of Sex Trafficking (Risk Factors)

**Figure 1:**
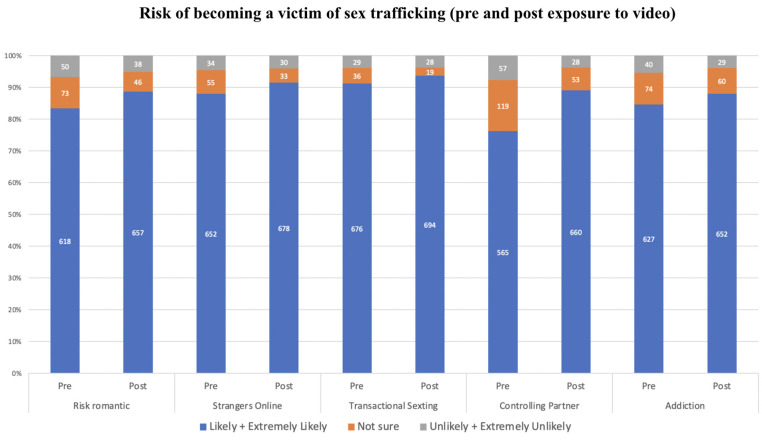
Risk factors.

Figure 1 describes the respondents’ perceptions of the likelihood that the situations presented in the videos (risk factors for human trafficking) could put a child at increased risk of becoming a victim of this crime. Before watching the videos, 76.2% of respondents reported that all situations presented in the videos were likely or extremely likely to put a child at risk of becoming a victim of sex trafficking. After watching the videos, we observed an increase in the composite score of all risk factors (Wilcoxon test statistic (W) = 89,959, *p* < 0.001). More specifically, after watching the videos, respondents showed the greatest increase in their level of awareness, from 76.2% to 89.1%, for the situation of ‘A student being in a romantic relationship with a controlling partner’.

#### 3.4.5. Awareness of Situations Experienced by Students Being Potential Signs of Sex Trafficking (Signs)

**Figure 2:**
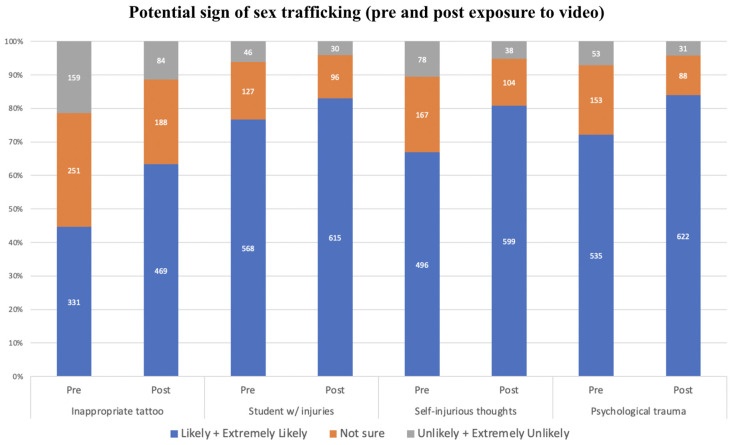
Signs of human trafficking.

Figure 2 describes the respondents’ perceptions of the likelihood that the situations presented in the videos could be signs of human trafficking. Before watching the videos, more than 40% of respondents responded with likely or extremely likely to all situations presented in the videos as being potential signs of sex trafficking. After watching the videos, we observed an increase in the composite risk factors score (W = 14,141, *p* <0.001). After watching the videos, their level of awareness increased for all potential signs of sex trafficking, with the largest increase observed from 44.7% to 63.3% for ‘A student with a tattoo with sexual content or a male name’.

#### 3.4.6. Open-Ended Question: Based on Your Experience, What Other Situations and Behaviors Can Put Youth at High Risk of Sex Trafficking?

Respondents reported, in open-ended questions, a series of situations that the research team grouped into eight themes: (1) familial vulnerability, (2) social vulnerability, (3) behavior changes, (4) personal safety and mental health, (5) socio-economic factors, (6) online behavior, (7) sexual vulnerability, and (8) substance misuse. The theme of familial vulnerability was named by 36% of respondents and included situations related to domestic or child abuse, neglect, lack of supervision, foster care, and general conflict at home. Social vulnerability was named by 31% of respondents and included social factors that leave a student more prone to exploitation such as peer pressure, bullying, insecurity, lack of supervision or support system, being groomed, a feeling of not belonging, and isolation, as examples. Changes in behavior were named by 29% of respondents and included changes in performance/attendance, as well as other behavioral indicators such as sensitive temperament, fatigue, startle response, or making risky social decisions. Personal safety and mental health issues were named by 27% of respondents and included trauma associated with domestic violence, homelessness, abuse, or neglect or being in foster care, as well as conditions such as depression or low self-worth that left a minor more vulnerable to exploitation. Socio-economic factors were named by 19% of respondents and included financial circumstances pushing students to engage in risky behaviors for money, either because they want to be financially independent of their family or because they want to help their family financially. Online behavior was named by 14% of respondents and included social media use, chat rooms, “catfishing”, (pretending to be a different age or a different person online), or being groomed online by strangers and adults. Sexual vulnerability was named by 12% of respondents and included developmentally inappropriate sexual behavior and unsafe or public underage sex, as well as being sexually harassed and sexually harassing others. It can also include sexual trauma history and making risky sexual decisions. Finally, substance misuse was named by 7% of respondents and included the use of drugs or alcohol by the parents, the child, or their friends.

#### 3.4.7. Awareness of the Different Modalities to Report a Potential Case of Sex Trafficking Involving a Minor

Participants were asked to select, from a pre-identified list, the most appropriate ways to report a potential case of sex trafficking involving a minor. Before watching the videos, 97.6% of participants selected one of the appropriate responses listed in the videos and 2.4% of participants responded, “I don’t know”. Immediately after watching the videos, the frequency of correct responses increased, with 98.8% of participants responding with one of the appropriate answers and 1.2% or 9 participants responding “I don’t know” (*p* = 0.039) (we considered participants who responded with either a given appropriate response or another response that was deemed appropriate as correct and participants who only responded with “I don’t know” as incorrect). We can conclude that respondents increased their awareness of reporting mechanisms as the proportion of respondents who responded with “I don’t know” after watching the videos was significantly less than those who responded “I don’t know” before watching the video.

#### 3.4.8. Open-Ended Question: If You Have Reported a Potential Case of Sex Trafficking, Please Describe What Prompted You to Report the Case and How You Reported It

Sixty-one respondents, corresponding to eight percent of the sample, described situations in which they reported a potential case of sexual exploitation. Fifty-one responses to this question could be grouped into the following themes: (1) change in a student’s behavior, (2) grooming indicators, and (3) physical indicators. The remaining 10 did not provide enough information to be coded.

Forty-four percent (*n* = 27) of respondents wrote that behavioral indicators prompted them to report a potential case of sex trafficking involving students. Such indicators included a change in a student’s behavior (including temperament, fatigue, and startle response), a change in school performance/attendance, signs of distress or mental health issues, awareness of the student being exposed to risky online behaviors, developmentally inappropriate sexual behavior, underage sex, and a student being sexually harassed or having sexually harassed others. Grooming indicators were reported by 25% (*n* = 15) of respondents and entailed circumstances where the reporter or a colleague noticed signs of grooming, including the student having an older boyfriend or older friends that they would be seen with or signs of control or inappropriate boundaries in a student’s relationship. Finally, 15% (*n* = 9) of respondents to this question reported that physical signs of abuse prompted them to report the case, with physical indicators including tattoo branding, new tattoos on a student, bruises/injuries, or changes in showing very little or a lot of skin.

#### 3.4.9. Determinants of Knowledge about Human Trafficking

Table 3 displays the predictors of respondent’s awareness of sex trafficking at baseline, based on each outcome variable described in Table 1. After conducting likelihood ratio tests (LRTs), it was determined that age, years of teaching/counseling experience, and level of education were all better suited as ordinal predictors rather than the categorical variables in the regression model.

For each increase in the level of work experience, respondents had 46% increased odds of being aware of situations that could put students at risk of becoming victims of sex trafficking (OR 1.46, 95% CI 1.15, 1.85). Additionally, female respondents had 97% increased odds of being aware of such situations compared to male respondents (OR 1.97, 95% CI 1.41, 2.75), and those receiving training in the past two years had 49% increased odds compared to those never trained (OR = 1.49, 95% CI 1.04, 2.14).

Female respondents had 66% increased odds of being aware of situations experienced by students being potential signs of sex trafficking compared to male respondents (OR 1.66, 95% CI 1.19, 2.31) while respondents receiving training in the past two years had 114% higher odds than those never trained (OR = 2.14, 95% CI 1.49, 3.08). For each increase in education level, respondents had 51% increased odds of being aware of being in a position at work to interact with students who may be victims of sex trafficking, (OR 1.51, 95% CI 1.18, 1.95). Additionally, mental health professionals and school counselors had 139% increased odds of being aware of findings themselves in such position compared to educators (OR 2.39, CI 1.29, 4.68) while those receiving training in the past two years and more than two years ago had 214% and 81% higher odds than those never trained (OR = 3.14, 95% CI 2.07, 4.84) and (OR = 1.81, 95% CI 1.07, 3.14), respectively. For the awareness of the different modalities to report a potential case of sex trafficking involving a minor endpoint, those receiving training in the past two years had 709% increased odds of being aware compared to those with no prior training.

The results of the Hosmer–Lemeshow goodness-of-fit tests for all five models indicate no evidence of poor fit (*p* > 0.05).

## 4. Discussion

The results of our study show that most school personnel have a basic understanding of the risk factors of abuse and most recognize that they could find themselves in a position to interact with potential victims of human trafficking. However, our data also indicate that training on human trafficking is lacking for these professionals, with more than half of respondents in our sample stating they never received training on this topic.

Some school personnel, like school counselors and school nurses, have a legal responsibility to report child sexual exploitation and, as such, play a critical role in prevention and intervention efforts [25]. Yet, even as mandated reporters, previous literature shows that these professionals may express reservations about reporting potential abuse, due to dissatisfaction with follow-up services delivered by child protection agencies [26]. The lack of trust in the response system coupled with a lack of awareness and response training may lead to school staff underreporting this serious crime. Furthermore, children, like adult victims, may find it challenging to self-recognize as victims [2]. Consequently, the responsibility falls on the adults with whom they interact. School personnel, who regularly interact with children, such as school teachers and counselors, hold a pivotal role in protecting and recognizing situations of abuse [5,6]. Exposure to brief educational videos, such as those tested in our study, may not be sufficient to provide the viewer with adequate knowledge to respond to a potential instance of human trafficking. However, it may increase basic knowledge about human trafficking and interest in attending educational initiatives examining this phenomenon more in depth. For example, in our study, it was clear that knowledge about commercial sex in minors being considered human trafficking was lacking and watching a video about this definition increased such knowledge. Our results also show the limitation of brief videos in establishing long-term memory with a significant decrease in the learners’ ability to retrieve the information gained 6 months after exposure to the videos. In the psychology of education, it is known that the retrieval of information involves processes used to recall memories of previous experiences [27]. These processes are initiated and directed by retrieval cues present in the learner’s surroundings (such as prompts, questions, or problems to solve) or within the learner’s mind (associations of thoughts or ideas) [25]. When such clues are not part of the learning process, or the learner has limited opportunities to use the knowledge acquired, long-term memory becomes challenging.

Our results indicate that there is a clear need for policy actions to enhance training opportunities that meet the educational needs of school personnel around this topic. We believe that there is also a need to develop response systems that lead to the protection of victims. Such systems need to be trusted by school personnel who are in a position to refer children to adequate support services. However, it is important to note that training without the creation of adequate response systems is useless and potentially harmful in stigmatizing individuals based on their vulnerabilities with a risk of criminalization of the victims.

Moreover, within the broader context of victimization and prevention efforts at large, several factors make children more susceptible to a variety of risks among which human trafficking is only one of them. Such factors include low socioeconomic status, family violence, child sexual abuse, mental health issues, and being in foster care or experiencing homelessness. These vulnerabilities, coupled with isolation and the influence of social media and gaming platforms as described in the videos, increase the likelihood that children would establish connections with adults grooming them into situations of abuse, exacerbating one’s risk for victimization [4,6]. This aspect was acknowledged by respondents in our survey, who identified socio-economic factors, unhealthy social and family relationships, and their online behaviors as a combination of primary risk factors for victimization.

### Limitations

This study presents several limitations. First, this study did not aim to evaluate the content of the Blue Campaign videos, and as such, we assumed the videos appropriately depict the correct way to handle specific situations experienced by youth. Second, for the pre- and post-study analysis, there was no control group included for comparison. Third, an increase in school personnel’s awareness about human trafficking does not mean that such knowledge is actionable or leads to better outcomes for the victim. Fourth, the results need to be interpreted based on the context and situations presented in the videos; it is important to note that risks of youth exploitation may be very different based on the local context, and awareness videos should reflect the nuance of such diversity. Finally, this evaluation is based on a pre- and post-study design conducted using crowdsourcing technology using their device to complete a series of questions and watch the videos, and it is reasonable to believe that in a different setting, knowledge retention could be greater.

## 5. Conclusions

The school personnel represented in our study showed a high level of awareness of risk factors for sex trafficking but less awareness of the definition of sex trafficking in children. Exposure to short educational videos was effective in increasing this specific basic knowledge. However, one-time exposure to a video may not lead to long-term knowledge retention as noted by the 6-month follow-up results. In addition, it is important to note that in the absence of adequate protocols to respond to a potential situation of human trafficking and mechanisms to refer the victim to adequate services specialized in this field, identification may result in unethical practices and cause harm to the victim.

## Figures and Tables

**Table 1:** Outcome variables.

Dependent Variable	Question	Answer Options and Coding
1. Awareness of commercial sex (sex in exchange of money or goods) involving minors being considered sex trafficking [variable labeled: definition]	A situation in which a person under the age of 18 is having sex in exchange for money or goods is:	A crime but not an instance of human trafficking (0) Not a crime (0) An instance of human trafficking (1) Binary outcome: 1 = A and B 0 = C
2. Awareness of situations that could potentially put students at risk of becoming a victim of sex trafficking * [variable labeled: risk factors]	Could any of the following situations—experienced by a student—put them at risk of becoming a victim of sex trafficking? Risk romantic: A student in a romantic relationship with someone who is noticeably older. Strangers online: A student under the age of 18 talking to strangers online. Transactional sexting: A student sexting with strangers in exchange for money Controlling partner: A student in a romantic relationship with a controlling partner Addiction: A student with substance abuse or addiction problems	Extremely unlikely (1) Unlikely (2) Not sure (3) Likely (4) Extremely likely (5) Binary outcome: 1 = the MFA factor score above the median 0 = the MFA factor score equal or below the median
3. Awareness of situations experienced by students that could potentially be signs of sex trafficking * [variable labeled: signs]	Could any of the following situations—experienced by a student—be a potential sign of sex trafficking? Inappropriate tattoo: A student with a tattoo with sexual content or a male name Student w/injuries: A student presenting with unexplained bruises or other physical injuries Self-injurious thoughts: A student with signs of self-harm and suicidal thoughts Psychological trauma: A student who is showing signs of psychological trauma (e.g., memory loss)	Extremely unlikely (1) Unlikely (2) Not sure (3) Likely (4) Extremely likely (5) Binary outcome: 1 = the MFA factor score above the median 0 = the MFA factor score equal/below the median
4. Awareness of being in a position at work to interact with students who are victims of sex trafficking [variable labeled: position]	At your job, do you think you could be in a position to interact with a student who may be a victim of sex trafficking?	No (0) I am not sure (1) Yes (2) Binary outcome: 1 = C 0 = A and B
5. Awareness of the different modalities to report a potential case sex trafficking involving a minor [variable labeled: reporting]	What are the appropriate ways to report a potential case of sex trafficking involving a minor? [check all that apply]	School’s protocol Call 911 Call ICE (Immigration and Customs Enforcement) Call the National Human Trafficking Line I do not know. Other (please specify) Binary outcome: 1 = A, B, C, D and F 0 = E

* This variable was computed as a factor score from a Multiple Factor Analysis (MFA) of the pre- and post-intervention data.

**Table 2:** Demographic characteristics of study population.

Baseline (*n* = 741)	*n* (%)
Age	
18–24	69 (9.3%)
25–34	200 (27.0%)
35–44	204 (27.5%)
45–54	155 (20.9%)
>54	113 (15.2%)
Sex *	
Female	425 (62.1%)
Male	259 (37.9%)
Race *	
White	528 (77.2%)
Non-white	151 (22.1%)
Prefer not to say	2 (0.3%)
Education	
Bachelor’s degree	274 (37.0%)
High school, GED, or some college	112 (15.1%)
Post-graduate degree (e.g., Master’s degree, PhD, MD etc.)	355 (47.9%)
Experience	
Less than 1 year	14 (1.9%)
1–5 years	164 (22.1%)
6–10 years	172 (23.2%)
More than 10 years	391 (52.8%)
Job Title	
Administrator	64 (8.6%)
Counselor	117 (15.8%)
Educator	454 (61.3%)
Mental Health Professional	22 (3.0%)
Other School Staff	84 (11.3%)
Census Region	
Midwest	156 (21.1%)
Northeast	162 (21.9%)
South	286 (38.6%)
West	137 (18.5%)
Received Training (Have you ever received specific training about sex trafficking?)	
Yes, within the last 2 years	231 (31.2%)
Yes, more than 2 years ago	91 (12.3%)
No	419 (56.5%)

* Information not available for 57 respondents. Note: Information on population reporting White race alone and population under 1.37 of the poverty thresholds could not be determined for 1 case and information on median household income could not be determined for 3 cases due to missing data from the AHRQ database.

**Table 3:** Results of logistic regression models.

	Outcomes				
Predictors	Awareness of Commercial Sex	Awareness of Situations That Could Potentially Put Students at Risk	Awareness of Situations Experienced by Students Being Potential Signs of Sex Trafficking	Awareness of Being in a Position at Work to Interact with Students Who Are Victims of Sex Trafficking	Awareness of the Different Modalities to Report a Potential Case of Sex Trafficking Involving A Minor
	OR (95% CI)	OR (95% CI)	OR (95% CI)	OR (95% CI)	OR (95% CI)
Intercept	0.56 (0.17, 1.89)	0.14 (0.04, 0.49)	0.20 (0.06, 0.68)	0.26 (0.07, 0.97)	2686.51 (37.37, 354,656.20)
Age	0.88 (0.75, 1.03)	1.04 (0.88, 1.22)	0.98 (0.84, 1.15)	0.91 (0.77, 1.08)	1.06 (0.65, 1.78)
Level of Education	1.20 (0.95, 1.52)	0.99 (0.79, 1.26)	0.86 (0.68, 1.08)	1.51 (1.18, 1.95) *	0.94 (0.45, 1.90)
Experience	1.22 (0.97, 1.53)	1.46 (1.15, 1.85) *	1.20 (0.96, 1.52)	1.19 (0.93, 1.52)	0.60 (0.25, 1.26)
Educator	Ref	Ref	Ref	Ref	Ref
Mental Health Professional or Counselor	1.01 (0.61, 1.68)	0.90 (0.54, 1.50)	1.01 (0.61, 1.67)	2.39 (1.29, 4.68) *	1.10 (0.26, 7.65)
Administrator or Other School staff	1.29 (0.87, 1.93)	1.09 (0.73, 1.63)	0.94 (0.63, 1.40)	1.40 (0.91, 2.16)	1.20 (0.35, 5.61)
Northeast	Ref	Ref	Ref	Ref	Ref
South	1.59 (0.83, 3.05)	1.06 (0.54, 2.08)	1.51 (0.78, 2.97)	1.55 (0.77, 3.11)	0.34 (0.03, 3.25)
Midwest	1.60 (0.81, 3.20)	1.02 (0.50, 2.06)	1.17 (0.58, 2.38)	1.80 (0.85, 3.78)	0.40 (0.03, 4.21)
West	1.54 (0.76, 3.13)	1.11 (0.54, 2.30)	1.47 (0.72, 3.07)	1.77 (0.82, 3.82)	0.82 (0.05, 12.66)
White	Ref	Ref	Ref	Ref	Ref
Non-White	0.75 (0.50, 1.11)	1.16 (0.77, 1.74)	0.87 (0.58, 1.30)	1.05 (0.68, 1.62)	1.37 (0.38, 6.61)
Male	Ref	Ref	Ref	Ref	Ref
Female	1.13 (0.81, 1.56)	1.97 (1.41, 2.75) *	1.66 (1.19, 2.31) *	1.03 (0.72, 1.48)	0.38 (0.08, 1.22)
Median Household Income	0.96 (0.74, 1.23)	0.91 (0.70, 1.17)	1.06 (0.82, 1.37)	0.91 (0.69, 1.19)	0.59 (0.27, 1.25)
Less than median percentage (≤79.5%) of the population reporting as of White race	Ref	Ref	Ref	Ref	Ref
More than median percentage (>79.5%) of the population reporting as of White race	0.95 (0.67, 1.35)	0.94 (0.66, 1.35)	1.18 (0.83, 1.68)	1.17 (0.80, 1.72)	1.33 (0.46, 3.91)
Percentage of the population under the 1.37 of the poverty thresholds	0.99 (0.97, 1.00)	1.01 (0.99, 1.02)	1.01 (0.99, 1.03)	1.00 (0.98, 1.02)	0.97 (0.94, 1.02)
Density of the population (≤2184.41 persons per square mile)	Ref	Ref	Ref	Ref	Ref
Density of the population (>2184.41 persons per square mile)	0.90 (0.45, 1.78)	0.91 (0.45, 1.85)	1.29 (0.64, 2.63)	0.83 (0.40, 1.72)	0.37 (0.03, 3.66)
Received training for sex trafficking—No	Ref	Ref	Ref	Ref	Ref
Yes, within last 2 years	1.18 (0.83, 1.70)	1.49 (1.04, 2.14) *	2.14 (1.49, 3.08) *	3.14 (2.07, 4.84) *	8.09 (1.55, 148.99) *
Yes, more than 2 years ago	1.33 (0.81, 2.22)	0.88 (0.53, 1.46)	1.59 (0.96, 2.61)	1.81 (1.07, 3.14) *	3.88 (0.72, 73.07)

* significant at *p* < 0.05 level.

## Data Availability

Data are available upon reasonable request.
